# Synthesis and Herbicidal Activity Evaluation of Novel β-Carboline Derivatives 

**DOI:** 10.3390/molecules17043969

**Published:** 2012-04-02

**Authors:** Qunfang Weng, Jingfei Huang, Yong Zeng, Yueye Deng, Meiying Hu

**Affiliations:** 1 Key Laboratory of Pesticide and Chemical Biology, Ministry of Education, Guangzhou 510642, China; Email: huabao@scau.edu.cn (Q.W.); huangjingfei1983@yahoo.com.cn (J.H.); yongz800@163.com (Y.Z.); dengyueye@163.com (Y.D.); 2 Laboratory of Insect Toxicology, South China Agricultural University, Guangzhou 510642, China

**Keywords:** *N'*-substituted-1,2,3,4-β-tetrahydro-β-carboline-3-carbohydrazide, β-carboline, *N'*-substituted-β-carboline-3-carbohydrazides, 1-substituted-β-carboline-3-carbohydrazides, herbicidal activity

## Abstract

Based on the original structure of harmine, several novel 1,2,3,4-tetrahydro-β-carboline, β-carboline and 1-substituted-β-carboline derivatives bearing a substituted carbohydrazide group at C-3 were designed and synthesized to investigate the structure-activity relationship of their analogues. All of the compounds were characterized by infrared (IR), proton and carbon nuclear magnetic resonance (^1^H-NMR, ^13^C-NMR), and mass spectroscopy (MS). The bioassay tests showed that *N'*-benzylidene-1-phenyl-β-carboline-3-carbohydrazide (C_25_H_18_N_4_O, m.w. 390.4) (**c2**) and *N'*-(4-trifluoromethyl-benzylidene)-1-phenyl-β-carboline-3-carbohydrazide (C_26_H_17_N_4_OF_3_, m.w. 458) (**d2**) exhibited good inhibitory activity against dicotyledonous and monocotyledonous weeds, with EC_50_ values of 4.83 µM and 14.25 µM, respectively.

## 1. Introduction

Harmine compounds and their structurally related compounds, belonging to the β-carboline alkaloids class, are present in medicinal plants such as *Peganum harmala* and *Eurycoma longifolia*. These compounds have recently drawn increasing interest due to their diverse biological activities, including pharmacological, neurophysiologic and biochemical activities [1,2,3,4,5,6]. Some plants which contain these compounds are used in traditional medicine in China, Brazil and other areas of the World for their emmenagogue, abortifacient, anticancer, antispasmodic and sedative effects [7,8].

As for pest management, the extracts of *Peganum L*. plant species containing a mixture of harmine, harmaline and norharman, as well as their derivatives, have long been proven to have insecticidal, fungicidal and plant growth regulatory properties [9,10,11,12,13,14]. Some research has revealed that *P. harmala* residues could cause great negative effects on seedling length, seedling dry weight, leaf area and chlorophyll content of *Avena fatua* L. (Poaceae) and *Convolvulus arvensis* L. (Convolvulaceae), showing their potential herbicidal activity [15,16], but there has been little systematic research on the effect of substituents on the pesticidal activities, therefore, the present study was designed to synthesize a series of novel β-carboline derivatives bearing a substituted carbohydrazide group at C-3, followed by their *in vivo* herbicidal activity evaluation.

## 2. Results and Discussion

### 2.1. Chemistry

**Scheme 1:**
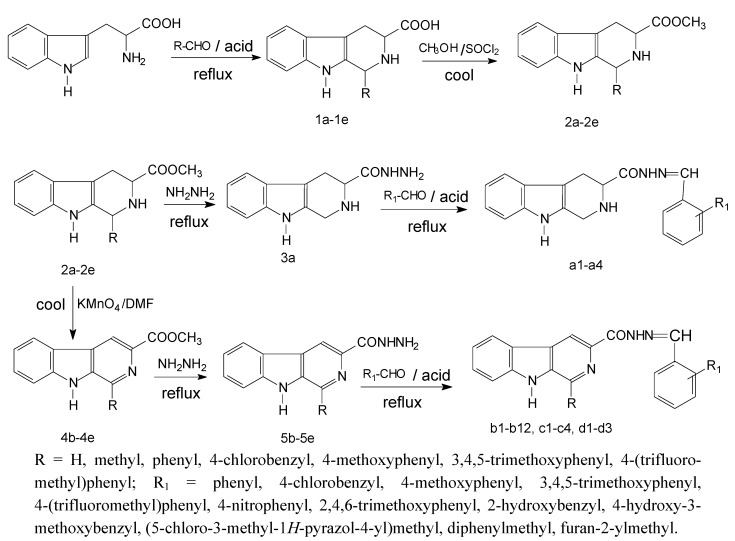
The synthetic protocols for the target compounds.

Previous structure-activity relationship studies had demonstrated the influence of substituents in positions-1, -3, and -9 of the β-carboline skeleton for a variety of synthetic β-carboline derivatives [17,18,19,20]. In order to study the effect of main structure and the substituent groups at position-1 and -3 on their herbicidal activity, we synthesized a series of 23 novel 1,2,3,4-tetrahydro-β-carboline and β-carboline derivatives bearing a substituted carbohydrazide group at C-3 and substituted groups at C-1 according to Love [17] (Scheme 1). According to their structural characteristics, these compounds could be divided into four series, including four *N'*-substituted-1,2,3,4-tetrahydro-β-carboline-3-carbohydrazides, 12 *N'*-substituted-β-carboline-3-carbohydrazides, four *N'*-benzylidene-1-substituted-β-carboline-3-carbohydrazides and three N'-substituted-benzylidene-1-substituted-β-carboline-3-carbohydrazides. All these compounds were characterized by their melting point, mass, infrared, ^1^H-NMR and ^13^C-NMR spectra that confirmed the proposed structures of the new compounds. It was also found that the Pictet-Spengler reactions of L-tryptophan with different aldehydes were different, as the reaction rates were not only related with the size of aldehydes, but also with the electronic properties of their substituents. Thus, 1,2,3,4-tetrahydro-β-carboline could be produced by the reaction of paraformaldehyde with L-tryptophan under anhydrous conditions. Benzaldehydes with an electron-withdrawing substituent reacted more easily with L-tryptophan than those bearing an electron-donating substituent. In this study, under the conditions of elevated temperature and increased pressure, condensation of tetrahydro-β-carboline and β-carboline with aromatic aldehydes in the presence of dehydrating agents under reflux in ethanol yielded the corresponding carbohydrazides. Because of the instability of tetrahydro-β-carbolines, they were hard to react with aromatic aldehydes, especially those aldehydes with electron-withdrawing substituents or high steric hindrance.

### 2.2. Herbicidal Activity

The target compounds were screened for herbicidal activities against rape and barnyard grass. The results (Table 1) indicated that these compounds showed some degree of herbicidal activities against rape, but weak activity against barnyard grass.

**Table 1:** Herbicidal activity of compounds (% I; concentration, μg/mL).

Compound	*B. campestris*		*E. crusgalli*	
	100	10	100	10
a1	25.31	3.75	15.04	0.00
a2	37.12	8.69	8.62	0.00
a3	33.54	5.28	23.80	2.51
a4	16.45	0.00	0.00	0.00
b1	55.63	18.13	35.94	11.83
b2	71.59	24.72	29.55	17.48
b3	58.40	16.27	37.12	15.07
b4	42.66	13.85	12.63	0.00
b5	47.32	18.02	0.00	0.00
b6	85.41	30.95	43.19	24.65
b7	50.13	19.36	47.61	18.05
b8	63.25	27.44	32.95	13.26
b9	17.85	2.59	0.00	0.00
b10	90.83	32.17	28.05	6.34
b11	23.68	7.52	0.00	0.00
b12	10.46	0.00	0.00	0.00
c1	87.05	37.22	51.47	21.85
c2	100	62.09	73.93	30.38
c3	82.35	27.12	31.16	19.35
c4	91.73	66.97	43.72	16.74
d1	76.49	35.38	35.68	9.76
d2	100	57.85	60.32	27.94
d3	93.71	66.90	57.87	24.15
harmine	53.71	20.16	32.49	10.82
2,4-D	100	—	100	100

**Figure 1:**
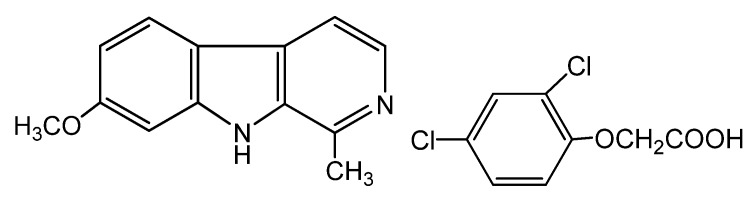
The structural formula of harmine and 2,4-D [(2,4-dichlorophenoxy)acetic acid].

Their herbicidal activities were determined by the core structure and the substituents at positions 1 and 3. The fully aromatic β-carbolines showed better herbicidal activities than those with tetrahydro-β-carboline moieties and the appropriate substituents at position 1 which could reinforce their activities. In this research, it was found substituted phenyls with an electron-withdrawing substituent and little steric hindrance had better activities. The substituents of position 3 could also affect the activity. An electron-withdrawing substituent or substructure with a high herbicidal activity could enhance their bioactivities. Furthermore, it was found that some seeds would not germinate after treatment with compounds at the concentration of 100 μg/mL. On the other hand, the roots of the control treated with 2,4-D (a common herbicide, Figure 1) were inhibited after germinating. According to the primary screening results, two compounds—**c2** and **d2**—with higher activity were selected for further investigations of EC_50_ and probit (mortality%)-log (concentration) lines. The results are shown in Table 2.

**Table 2:** EC_50_ values of compounds **c2** and **d2**.

Compound	y = a + bx	EC_50_
c2	y = 4.4025 + 0.8710x	4.83
d2	y = 2.5709 + 2.1052x	14.25

## 3. Experimental

### 3.1. General

All reagents were purchased from commercial suppliers and dried and puriﬁed when necessary. Melting points were determined on an XT-4 binocular microscope (Beijing Tech Instrument Co., Beijing, China) and were uncorrected. ^1^H-NMR spectra were recorded in DMSO-*d_6_* solutions with a Bruker AC-P500 (500 MHz) instrument, using tetramethylsilane as an internal standard. Infrared spectra were measured on an ACATAR 360 Fourier Transform Infrared Spectrometer using potassium bromide (KBr) disks, scanning from 400 to 4,000 cm^−1^. MS were obtained on an Agilent 6890N-5975 instrument (Agilent Technologies, Inc., Palo Alto, CA, USA). Elemental analyses (C, H and N) were carried out on an Elementar Vario EL CHNS Elemental Analyzer (Elementar, Hanau, Germany). FAB-MS spectra were obtained from VG ZAB-HS spectrometer (VG Instrument Inc., Manchester, UK). Silica gel F254 was used in analytical thin-layer chromatography (TLC) and silica gel was used in column chromatography. Yields were not optimized.

#### 3.1.1. General Synthesis

The synthetic route for the preparation of 1,2,3,4-tetrahydro-β-carboline-3-carbohydrazide **3a** and β-carboline-3-carbohydrazides **5a**–**e** is presented in Scheme 1. Methyl tetrahydro-β-carboline-3-carboxylates **2a**–**e** were prepared through Pictet-Spengler condensation of L-tryptophan (**1**) with polyoxymethylene (**a**), acetaldehyde (**b**), 3,4,5-trimethoxybenzaldehyde (**c**), 4-(trifluoromethyl) benzaldehyde (**d**), and benzaldehyde (**e**), in acid media, and subsequent esterification of the corresponding carboxylic acids with methanol and thionyl chloride. Oxidation of methyl 1,2,3,4-tetrahydro-β-carboline-3-carboxylates **2a**–**e** with KMnO_4_ under cooled DMF, furnished methyl β-carboline-3-carboxylates **4b**–**e**. Conversion of **2a** and **4b**–**e** to 1,2,3,4-tetrahydro-β-carboline-3-carbohydrazides **3a** and β-carboline-3-carbohydrazides **5a**–**e** was carried out by reaction with hydrazine hydrate in ethanol under reflux according to the procedures described in literature for similar compounds [21]. Condensation of **3a** and **5b**–**e** with the aromatic aldehydes 4-methoxybenzaldehyde, 4-nitrobenzaldehyde, 3,4,5-trimethoxybenzaldehyde, 2,4,6-trimethoxybenzaldehyde, 2-hydroxybenzaldehyde, 4-hydroxy-3-methoxybenzaldehyde, benzaldehyde, and 4-chlorobenzaldehyde under reflux in ethanol yielded the carbohydrazides **a1**–**4**, **b1**–**12**, **c1**–**4** and **d1**–**3**.

*General Synthesis Procedure for* N'-*Substituted-1,2,3,4-tetrahydro-β-carboline-3-carbohydrazides* **a1**–**4**, N-*Substituted-β-carboline-3-carbohydrazides* **b1**–**12**, *1-Substituted-*N-*phenyl-β-carboline-3-carbohydrazides* **c1**–**4** *and 1-Substituted-*N-*substituted-β-carboline-3-carbohydrazides* **d1**–**3**

A solution of **3a** derivatives **5b**–**e** (1 mmol) in ethanol (15 mL) was stirred and reﬂuxed for 10 min until complete dissolution. Then, a solution of aldehyde (1 mmol) in ethanol (3 mL) was added. Five drops of acetic acid were added as a catalyst. The mixture was reﬂuxed for 0.5 h–4 h. Precipitates were ﬁltered and dried, furnishing the title compounds **a1**–**4** (**b1**–**12**, **c1**–**4** and **d1**–**3**) in 62–90% yields.

#### 3.1.2. Spectral Data

N'*-Benzylidene-1*,*2*,*3*,*4-tetrahydro-β-carboline-3-carbohydrazide* (C_19_H_18_N_4_O, m.w. 318.4) (**a1**). Yield: 83.5%; m.p.: 247–249 °C; FAB-MS *m/z* (M+1) 319; IR (cm^−1^): 3409, 2923, 1675, 1625; ^1^H-NMR: 2.81 (1H, q, *J* = 12.5 Hz, C(4)H, c), 3.12 (1H, dd, *J* = 18 Hz, C(4)H, c), 3.62 (1H, d, *J* = 13.5 Hz, C(1)H, c), 3.68 (1H, dd, *J* = 14.5 Hz, N(2)H, c), 3.83 (1H, d, *J* = 14 Hz, C(1)H, c), 5.55 (1H, dd, *J* = 2 Hz, C(3)H, c), 6.97 (9H, m, Ph), 8.98 (1H, s, NH), 10.77 (1H, s, N(9)H, c); ^13^C-NMR: 25.6, 32.1, 75.2, 103.5, 109.7, 117.3, 118.8, 120.5, 126.4, 127.9, 128.7, 130.2, 132.7, 133.7, 138.4, 147.2, 160.3; Anal. Calcd for C_19_H_18_N_4_O: C, 71.68; H, 5.70; N, 17.60; Found: C, 71.35; H, 5.92; N, 17.53.

N'*-(4-Methoxybenzylidene)-1*,*2*,*3*,*4-tetrahydro-β-carboline-3-carbohydrazide* (C_20_H_20_N_4_O_2_, m.w. 348.4) (**a2**). Yield: 93%; m.p.: 258–259 °C; FAB-MS *m/z* (M+1) 349; IR (cm^−1^): 3425, 2923, 2851, 1720, 1607, 1508, 1250, 1164, 1028; ^1^H-NMR: 2.77 (1H, q, *J* = 25 Hz, C(4)H, c), 3.10 (1H, dd, *J* = 18 Hz, C(4)H, c), 3.61 (2H, d, *J* = 14 Hz, C(1)H, c), 3.80 (1H, bs, N(2)H, c), 3.83 (3H, s, Ph-OCH_3_), 5.45 (1H, dd, *J* = 2 Hz, C(3)H, c), 6.95–7.82(8H, m, Ph), 8.63(1 H, s, N=CH), 8.88 (1H, s, NH), 10.76 (1H, s, N(9)H, c); ^13^C-NMR: 25.8, 33.4, 55.6, 75.2, 103.5, 109.7, 115.2, 117.3, 118.8, 120.5, 126.4, 128.7, 130.2, 131.5, 133.7, 138.4, 160.1, 161.9; Anal. Calcd for C_20_H_20_N_4_O_2_: C, 68.95; H, 5.79; N, 16.08; Found: C, 68.71; H, 5.84; N, 15.96.

N'*-(3*,*4*,*5-Trimethoxybenzylidene)-1*,*2*,*3*,*4-tetrahydro-β-carboline-3-carbohydrazide* (C_22_H_24_N_4_O_4_, m.w. 408.5) (**a3**). Yield: 87%; m.p.: 207–209 °C; FAB-MS *m/z* (M+1) 409; IR (cm^−1^): 3375, 2964, 2919, 1697, 1584, 1458, 1327, 1128; ^1^H-NMR: 2.80 (1H, q, *J* = 25 Hz, C(4)H, c), 3.12(1H, dd, *J* = 17.5 Hz, C(4)H, c), 3.76 (9H, s, Ph-OCH_3_), 3.84 (1H, bs, N(2)H, c), 3.86 (2H, s, C(1)H, c), 5.45 (1H, dd, *J* = 1.5 Hz, C(3)H, c), 6.89 (6H, m, Ph), 8.66 (1H, s, N=CH), 8.93 (1H, bs, NH), 10.78 (1H, s, N(9)H, c); ^13^C-NMR: 26.3, 32.9, 56.4, 62.5, 74.2, 106.7, 107.5, 112.8, 120.4, 120.9, 122.1, 127.3, 128.5, 135.6, 137.2, 141.9, 148.1, 160.5, 161.8; Anal. Calcd for C_22_H_24_N_4_O_4_: C, 64.69; H, 5.92; N, 13.72; Found: C, 64.57; H, 6.01; N, 13.65.

N'*-(1*,*2*,*3*,*4-Tetrahydro-β-carboline-3-carbonyl)-N-(4-(trifluoromethyl)phenyl)formohydrazonamide* (C_20_H_18_F_3_N_5_O, 401.4) (**a4**). Yield: 62%; m.p.: 245–247 °C; FAB-MS *m/z* (M+1) 402; IR (KBr, cm^−1^): 3317, 2878, 1643, 1607, 1444, 1327, 1223, 1146; ^1^H-NMR: 2.66–2.68 (1H, q, *J* = 9.5 Hz, C(4)H, c), 2.78 (1H, dd, *J* = 18.5 Hz, C(4)H, c), 3.40 (1H, dd, *J* = 14 Hz, C(3)H, c), 3.86–3.90 (1H, d, *J* = 16 Hz, C(1)H, c), 3.94 (1H, d, *J* = 16.5 Hz, C(1)H, c), 4.26 (2H, bs, N(2)H, NH), 6.91–7.35 (8H, m, Ph), 9.05 (1H, s, NH), 10.68 (H, s, N(9)H, c); ^13^C-NMR: 25.7, 32.1, 73.9, 104.2, 112.1, 116.4, 118.2, 120.3, 121.8, 124.5, 126.1, 126.9, 128.6, 130.5, 136.2, 146.3, 148.7, 158.2; Anal. Calcd for C_20_H_18_F_3_N_5_O: C, 59.85; H, 4.52; N, 17.45; Found: C, 59.74; H, 4.68; N, 17.31.

N'*-Benzylidene-β-carboline-3-carbohydrazide* (C_19_H_14_N_4_O, m.w. 314.3) (**b1**). Yield: 89%; m.p.: 289–291 °C; FAB-MS *m/z* (M+1) 315; IR (cm^−1^): 3293, 1658, 1620, 1524; ^1^H-NMR: 7.31 (1H, t, *J* = 15 Hz, C(6)H, c), 7.45 (3H, m, *J* = 25.5 Hz, Ph(3,4,5)), 7.60 (1H, t, *J* = 15 Hz, C(7)H, c), 7.68 (1H, d, *J* = 8 Hz, C(8)H, c), 7.74 (2H, d, *J* = 8 Hz, PH(2,6)), 8.45 (1H, d, *J* = 8 Hz, C(5)H, c), 8.67 (1H, s, N=CH), 8.96 (1H, s, C(4)H, c), 8.98 (1H, s, C(1)H, c), 11.73–11.75 (1H, bs, –N(9)H, c), 12.07 (1H, s, –NH–N); ^13^C-NMR: 108.3, 114.6, 118.2, 120.8, 121.8, 121.9, 127.5, 128.4, 130.3, 130.5, 133.1, 135.3, 138.2, 141.4, 148.5, 161.9; Anal. Calcd for C_19_H_14_N_4_O: C, 72.60; H, 4.49; N, 17.82; Found: C, 72.48; H, 4.62; N, 17.69.

N'*-(4-Methoxybenzylidene)-β-carboline-3-carbohydrazide *(C_20_H_16_N_4_O_2_, m.w. 344.4) (**b2**). Yield: 84%; m.p.: 273–275 °C; FAB-MS *m/z* (M+1) 345; IR (cm^−1^): 3447, 3409, 2925, 1658, 1604, 1251; ^1^H-NMR: 3.82 (3H, s, Ph(4)-OCH_3_), 7.03 (2H, d, *J* = 10.5 Hz, Ph(3,5)), 7.31–7.34 (1H, t, *J* = 14.5 Hz, C(6)H, c), 7.60 (1H, t, *J* = 16 Hz, C(7)H, c), 7.68 (3H, d, *J* = 8.5 Hz, C(8)H, c; Ph(2,6)), 8.44 (1H, d, *J* = 7.5 Hz, C(5)H, c), 8.60 (1H, s, N=CH), 8.95 (1H, s, C(4)H, c), 8.96 (1H, s, C(1)H, c), 11.93 (1H, bs, –N(9)H, c), 12.01(1H, s, –NH–N); ^13^C-NMR: 55.8, 108.1, 113.5, 114.9, 118.4, 120.2, 121.7, 122.3, 127.6, 131.4, 133.8, 135.3, 138.1, 141.6, 149.2, 160.3, 160.9; Anal. Calcd for C_20_H_16_N_4_O_2_: C, 69.76; H, 4.68; N, 16.27; Found: C, 69.61; H, 4.86; N, 16.12.

N'*-(3*,*4*,*5-Trimethoxybenzylidene)-β-carboline-3-carbohydrazide* (C_22_H_20_N_4_O_4_, m.w. 404.4) (**b3**). Yield: 83%; m.p.: 304–306 °C; FAB-MS *m/z* (M+1) 405; IR (cm^−1^): 3430, 3239, 2922, 1661, 1619, 1532; ^1^H-NMR: 3.72 (3H, s, Ph(4)-OCH_3_), 3.86 (6H, d, *J* = 4.5 Hz, Ph(3,5)-OCH_3_), 7.03 (2H, d, *J* = 10.5 Hz, Ph(2,6)), 7.31 (1H, t, *J* = 15 Hz, C(6)H, c), 7.60 (1H, t, *J* = 15.5 Hz, C(7)H, c), 7.68 (1H, d, *J* = 8 Hz, C(5)H, c), 8.45 (1H, d, *J* = 7.5 Hz, C(8)H, c), 8.55 (1H, s, C(4)H, c), 8.58 (1H, s, C(1)H, c), 8.96 (1H, s, N=CH), 11.76 (1H, bs, –N(9)H, c), 12.03(1H, s, –NH–N); ^13^C-NMR: 56.5, 62.3, 107.2, 109.6, 114.4, 118.7, 120.4, 121.2, 121.9, 128.6, 130.1, 133.5, 134.8, 139.2, 141.6, 145.3, 148.9, 158.3, 161.7; Anal. Calcd for C_22_H_20_N_4_O_4_: C, 65.34; H, 4.98; N, 13.85; Found: C, 65.19; H, 5.24; N, 13.57.

N'*-(2*,*4*,*6-Trimethoxybenzylidene)-β-carboline-3-carbohydrazide* (C_22_H_20_N_4_O_4_, m.w. 404.4) (**b4**). Yield: 54%; m.p.: 213–215 °C; FAB-MS *m/z* (M+1) 405; IR (cm^−1^): 3268, 2920, 2854, 1669, 1605, 1227; ^1^H-NMR: 3.82 (6H, s, Ph(2,6)-OCH_3_), 3.84 (3H, s, Ph(4)-OCH_3_), 6.30 (2H, s, Ph(3,5)), 7.30 (1H, t, *J* = 15 Hz, C(6)H, c), 7.59 (1H, t, *J* = 15.5 Hz, C(7)H, c), 7.67 (1H, d, *J* = 8 Hz, C(5)H, c), 8.43 (1H, d, *J* = 8 Hz, C(8)H, c), 8.63 (1H, s, C(4)H, c), 8.90 (1H, s, C(1)H, c), 8.93 (1H, s, N=CH), 11.69 (1H, bs, –N(9)H, c), 11.98(1H, s, –NH–N); ^13^C-NMR: 55.7, 55.9, 95.2, 105.4, 108.6, 112.5, 118.3, 120.7, 121.6, 123.1, 127.9, 134.5, 134.8, 139.5, 141.2, 145.3, 148.9, 160.1, 161.5; Anal. Calcd for C_22_H_20_N_4_O_4_: C, 65.34; H, 4.98; N, 13.85; Found: C, 65.21; H, 5.19; N, 13.61.

N'*-(4-Chlorobenzylidene)-β-carboline-3-carbohydrazide* (C_19_H_13_ClN_4_O, m.w. 348.8) (**b5**). Yield: 93%; m.p.: 269–271 °C; FAB-MS *m/z* (M+1) 349; IR (cm^−1^): 3272, 1679, 1626, 1494; ^1^H-NMR: 7.31 (1H, t, *J* = 15 Hz, C(6)H, c), 7.53 (2H, d, *J* = 8.5 Hz, Ph(3,5)), 7.60 (1H, t, *J* = 16 Hz, C(7)H, c), 7.68 (1H, d, *J* = 8 Hz, C(5)H, c), 7.75 (2H, d, *J* = 8.5 Hz, Ph(2,6)), 8.45 (1H, d, *J* = 8 Hz, C(8)H, c), 8.67 (1H, s, C(4)H, c), 8.96 (1H, s, C(1)H, c), 8.98 (1H, s, N=CH), 11.762 (1H, bs, –N(9)H, c), 12.03(1H, s, –NH–N); ^13^C-NMR: 109.2, 113.8, 118.6, 121.1, 121.9, 123.1, 127.9, 130.4, 131.7, 134.1, 134.6, 137.8, 138.3, 139.5, 145.3, 148.9, 160.1; Anal. Calcd for C_19_H_13_ClN_4_O: C, 65.43; H, 3.76; N, 16.06; Found: C, 65.28; H, 3.95; N, 15.87.

N'*-(4-Nitrobenzylidene)-β-carboline-3-carbohydrazide* (C_19_H_13_N_5_O_3_, m.w. 359.3) (**b6**). Yield: 92%; m.p.: >300 °C; FAB-MS *m/z* (M+1) 360; IR (cm^−1^): 3446, 3230, 1684, 1627, 1518; ^1^H-NMR: 7.32 (1H, t, *J* = 15.5 Hz, C(6)H, c), 7.61 (1H, t, *J* = 15 Hz, C(7)H, c), 7.68 (1H, d, *J* = 8.5 Hz, C(5)H, c), 7.99 (2H, d, *J* = 8.5 Hz, Ph(2,6)), 8.32 (2H, d, *J* = 9 Hz, Ph(3,5)), 8.46 (1H, d, *J* = 8 Hz, C(8)H, c), 8.79 (1H, s, C(4)H, c), 8.97 (1H, s, C(1)H, c), 9.00 (1H, s, N=CH), 12.04 (1H, bs, –N(9)H, c), 12.40 (1H, s, –NH–N); ^13^C-NMR: 108.7, 114.1, 119.2, 120.6, 121.3, 123.7, 128.2, 134.2, 134.7, 134.6, 137.8, 139.5, 141.7, 145.4, 147.6, 158.3, 161.2; Anal. Calcd for C_19_H_13_N_5_O_3_: C, 63.51; H, 3.65; N, 19.49; Found: C, 63.36; H, 3.83; N, 19.21.

N'*-(2-Hydroxybenzylidene)-β-carboline-3-carbohydrazide *(C_19_H_14_N_4_O_2_, m.w. 330.3) (**b7**). Yield: 83%; m.p.: 283–285 °C; FAB-MS *m/z* (M+1) 331; IR (cm^−1^): 3457, 3254, 1663, 1620, 1493, 1351; ^1^H-NMR: 6.93 (2H, q, *J* = 10.5 Hz, Ph(3,5)), 7.30 (2H, m, *J* = 23.5 Hz, C(6)H, c; Ph(4)), 7.46 (1H, d, *J* = 7.5 Hz, C(5)H, c), 7.59 (1H, m, *J* = 32 Hz, Ph(6)), 7.69 (1H, t, *J* = 8.5 Hz, C(7)H, c), 8.44 (1H, d, *J* = 7.5 Hz, C(8)H, c), 8.85 (1H, s, C(4)H, c), 8.97 (1H, s, C(1)H, c), 8.98 (1H, s, N=CH), 11.62 (1H, bs, –N(9)H, c), 12.05(1H, s, –NH–N), 12.45 (1H, s, Ph-OH); ^13^C-NMR: 108.6, 114.3, 118.9, 120.1, 120.4, 120.9, 121.5, 123.8, 125.3, 127.6, 134.2, 135.1, 135.7, 138.3, 139.6, 145.2, 147.8, 160.3, 160.7; Anal. Calcd for C_19_H_14_N_4_O_2_: C, 69.08; H, 4.27; N, 16.96; Found: C, 68.94; H, 4.58; N, 16.72.

N'*-(4-Hydroxy-3-methoxybenzylidene)-β-carboline-3-carbohydrazide *(C_20_H_16_N_4_O_3_, m.w. 360.4) (**b8**). Yield: 83%; m.p.: 163–165 °C; FAB-MS *m/z *(M+1) 361; IR (cm^−1^): 3430, 2924, 1663, 1600, 1505, 1380, 1278; ^1^H-NMR: 3.85 (3H, s, Ph(4)-OCH_3_), 6.85 (1H, d, *J* = 8 Hz, Ph(5)), 7.08 (1H, t, *J* = 16.5 Hz, C(6)H, c), 7.31 (2H, m, *J* = 18 Hz, Ph(2,6)), 7.60 (1H, d, *J* = 23.5 Hz, C(5)H, c), 7.67 (1H, t, *J* = 8 Hz, C(7)H, c), 8.44 (1H, d, *J* = 7.5 Hz, C(8)H, c), 8.54 (1H, s, C(4)H, c), 8.94 (1H, s, C(1)H, c), 8.95 (1H, s, N=CH), 9.54 (1H, bs, Ph-OH), 11.74 (1H, bs, –N(9)H, c), 12.01 (1H, s, –NH–N); ^13^C-NMR: 56.4, 109.2, 114.1, 114.8, 119.2, 119.9, 120.6, 122.1, 123.5, 126.7, 128.3, 131.9, 135.4, 138.3, 139.6, 145.2, 147.6, 148.1, 150.8, 160.3; Anal. Calcd for C_20_H_16_N_4_O_3_: C, 66.66; H, 4.48; N, 15.55; Found: C, 66.38; H, 4.65; N, 15.29.

N'*-(Furan-2-ylmethylene)-β-carboline-3-carbohydrazide* (C_17_H_12_N_4_O_2_, m.w. 304.3) (**b9**). Yield: 61%; m.p.: >300 °C; FAB-MS *m/z *(M+1) 305; IR (cm^−1^): 3284, 1658, 1622; ^1^H-NMR: 6.64 (1H, t, *J* = 3 Hz, furan(4)), 6.90 (1H, d, *J* = 3.5 Hz, furan(3)), 7.30 (1H, t, *J* = 15 Hz, C(6)H, c), 7.61 (1H, d, *J* = 8 Hz, furan(5)), 7.67 (1H, t, *J* = 8 Hz, C(7)H, c), 7.86 (1H, s, C(4)H, c), 8.44 (1H, d, *J* = 8 Hz, C(8)H, c), 8.58 (1H, s, C(1)H, c), 8.95 (1H, d, *J* = 5 Hz, C(5)H, c), 8.97 (1H, s, N=CH), 11.74 (1H, d, *J* = 15 Hz, –N(9)H, c), 12.02 (1H, s, ‑NH–N); ^13^C-NMR: 108.7, 114.5, 115.8, 118.4, 119.3, 120.6, 122.1, 123.5, 128.3, 135.4, 137.6, 138.6, 139.6, 145.5, 148.3, 150.8, 160.4; Anal. Calcd for C_17_H_12_N_4_O_2_: C, 67.10; H, 3.97; N, 18.41; Found: C, 66.85; H, 4.18; N, 18.27.

N'*-((5-Chloro-3-methyl-1H-pyrazol-4-yl)methylene)-β-carboline-3-carbohydrazide* (C_17_H_13_ClN_6_O, m.w. 352.8) (**b10**). Yield: 74%; m.p.: 259–261 °C; FAB-MS *m/z* (M+1) 353; IR (cm^−1^): 3430, 3239, 2922, 1661, 1619, 1532; ^1^H-NMR: 2.50 (3H, s, pyrazole-CH_3_), 7.31 (1H, q, *J* = 15 Hz, C(6)H, c), 7.60 (1H, dd, *J* = 19.5 Hz, C(7)H, c), 7.67 (1H, d, *J* = 8 Hz, C(5)H, c), 8.43 (1H, d, *J* = 7.5 Hz, C(8)H, c), 8.57 (1H, s, N=CH), 8.91 (1H, s, C(4)H, c), 8.94 (1H, s, C(1)H, c), 11.69–11.71 (1H, bs, –N(9)H, c), 11.96 (1H, s, –NH–N), 13.18 (1H, bs, NH, pyrazole); ^13^C-NMR: 15.7, 107.2, 112.1, 112.8, 117.5, 119.6, 120.1, 124.7, 128.3, 134.6, 138.3, 138.6, 144.2, 145.6, 148.3, 149.8, 161.2; Anal. Calcd for C_17_H_13_ClN_6_O: C, 57.88; H, 3.71; N, 23.82; Found: C, 57.63; H, 3.96; N, 23.68.

N'*-(β-Carboline-3-carbonyl)-N-(4-(trifluoromethyl)phenyl)formohydrazonamide* (C_20_H_13_F_3_N_4_O, m.w. 382.3) (**b11**). Yield: 85%; m.p.: 248–250 °C; FAB-MS *m/z* (M+1) 398; IR (cm^−1^): 3248, 3222, 1647, 1617, 1337; ^1^H-NMR: 4.58 (1H, bs, N=C–NH), 6.92–7.02 (2H, m, Ph(2,6)), 7.20 (1H, q, *J* = 10 Hz, C(6)H, c), 7.30 (1H, d, *J* = 8 Hz, C(5)H, c), 7.39 (1H, dd, *J* = 10 Hz, C(7)H, c), 7.56 (3H, m, N=CH; Ph(3,5)), 8.38 (1H, d, *J* = 15 Hz, C(8)H, c), 8.81 (1H, s, C(4)H, c), 8.88 (1H, s, C(1)H, c), 11.64 (1H, bs, –N(9)H, c), 11.97 (1H, s, ‑NH–N); ^13^C-NMR: 109.6, 112.8, 117.5, 118.3, 119.6, 121.5, 124.5, 128.4, 128.9, 130.2, 131.8, 134.4, 134.8, 144.2, 147.6, 148.2, 150.3, 160.7; Anal. Calcd for C_20_H_13_F_3_N_4_O: C, 60.45; H, 3.55; N, 17.62; Found: C, 60.27; H, 3.74; N, 17.39.

N'*-(Diphenylmethylene)-β-carboline-3-carbohydrazide* (C_25_H_18_N_4_O, m.w. 390.4) (**b12**). Yield: 70%; m.p.: >300 °C; FAB-MS *m/z* (M+1) 391; IR (cm^−1^): 3257, 3175, 1667, 1624, 1495, 1254; ^1^H-NMR: 7.31 (1H, t, *J* = 10 Hz, C(6)H, c), 7.43 (5H, m, Ph_1_(2,3,4,5,6)), 7.60 (5H, m, Ph_2_(2,3,4,5,6)), 7.64 (1H, d, *J* = 8.5 Hz, C(5)H, c), 7.71 (1H, t, *J* = 10.5 Hz, C(7)H, c), 8.43 (1H, d, *J* = 7.5 Hz, C(8)H, c), 8.64 (1H, s, C(4)H, c), 8.96 (1H, s, C(1)H, c), 11.03 (1H, bs, –N(9)H, c), 12.04(1H, s, –NH–N); ^13^C-NMR: 108.4, 115.7, 118.1, 120.6, 121.4, 124.7, 128.3, 128.8, 131.2, 133.7, 134.1, 134.9, 138.3, 145.6, 148.6, 160.2, 160.8; Anal. Calcd for C_25_H_18_N_4_O: C, 76.91; H, 4.65; N, 14.35; Found: C, 76.68; H, 4.82; N, 14.16.

N'*-Benzylidene-1-methyl-β-carboline-3-carbohydrazide* (C_20_H_16_N_4_O, m.w. 328.4) (**c1**). Yield: 63%; m.p.: >300 °C; FAB-MS *m/z* (M+1) 329; IR (cm^−1^): 3275, 2921, 1667, 1623, 1523; ^1^H-NMR: 2.91 (3H, s, –CH_3_), 7.30 (1H, m, *J* = 16.2 Hz, C(6)H, c), 7.45 (1H, m, *J* = 8.4 Hz, C(7)H, c), 7.48 (2H, m, *J* = 14.4 Hz, C(3,5)H, Ph), 7.59 (1H, m, *J* = 16.2 Hz, C(4)H, Ph), 7.66 (1H, m, *J* = 8.4 Hz, C(8)H, c), 7.76 (2H, d, *J* = 8.4 Hz, C(2,6)H, Ph), 8.40 (1H, d, *J* = 7.8 Hz, C(5)H, c), 8.68 (1H, s, C(4)H, c), 8.80 (1H, s, –N=CH), 11.83 (1H, s, –NH, c), 12.03 (1H, s, –NH–N); ^13^C-NMR: 14.8, 111.5, 114.7, 119.6, 120.1, 124.7, 125.3, 128.8, 129.7, 134.2, 138.5, 139.6, 144.2, 145.6, 147.3, 148.7, 150.3, 159.8; Anal. Calcd for C_20_H_16_N_4_O: C, 73.15; H, 4.91; N, 17.06; Found: C, 72.98; H, 5.06; N, 16.85.

N'*-Benzylidene-1-phenyl-β-carboline-3-carbohydrazide* (C_25_H_18_N_4_O, m.w. 390.4) (**c2**). Yield: 87%; m.p.: 271–273 °C; FAB-MS *m/z* (M+1) 391; IR (cm^−1^): 3281, 3200, 1667, 1624, 1518; ^1^H-NMR: 7.37 (1H, m, *J* = 15.6 Hz, C(6)H, c), 7.46 (1H, m, *J* = 15.6 Hz, C(4)H, Ph_1_), 7.48 (2H, m, *J* = 15.6 Hz, C(3,5)H, Ph_1_), 7.60 (1H, m, *J* = 10.2 Hz, C(7)H, c), 7.62 (1H, m, *J* = 10.2 Hz, C(8)H, c), 7.70 (3H, m, *J* = 28.2 Hz, C(3,4,5)H, Ph_2_), 7.78 (2H, t, *J* = 8.4 Hz, C(2,6)H, Ph_1_), 8.22 (2H, m, *J* = 8.4 Hz, C(2,6)H, Ph_2_), 8.48 (1H, d, *J* = 7.8 Hz, C(4)H, c), 8.68 (1H, s, C(5)H, c), 8.97 (1H, s, –N = CH), 11.81 (1H, s, –NH, c), 11.93 (1H, s, –NH–N); ^13^C-NMR: 111.3, 116.1, 119.7, 121.4, 123.9, 126.5, 128.7, 128.9, 129.2, 129.5, 130.2, 130.8, 131.6, 132.1, 134.8, 136.5, 139.2, 142.7, 143.2, 149.3, 161.8; Anal. Calcd for C_25_H_18_N_4_O: C, 76.91; H, 4.65; N, 14.35; Found: C, 76.72; H, 4.84; N, 14.19.

N'*-Benzylidene-1-(4-(trifluoromethyl)phenyl)-β-carboline-3-carbohydrazide* (C_26_H_17_F_3_N_4_O, m.w. 458.4) (**c3**). Yield: 80.5%; m.p.: 235–237 °C; FAB-MS *m/z* (M+1) 459; IR (cm^−1^): 3436, 1730, 1622, 1325; ^1^H-NMR: 7.35–8.43 (13H, Ph1, Ph2, c), 8.67 (1H, s, C(4)H, c), 9.02 (1H, s, –N=CH), 11.87 (1H, s, –NH, c), 12.07 (1H, s, –NH–N); ^13^C-NMR: 112.5, 115.7, 120.1, 121.3, 122.9, 124.3, 127.3, 128.8, 129.1, 130.5, 131.2, 131.8, 134.7, 135.6, 138.5, 139.6, 141.6, 144.2, 147.3, 148.7, 160.8; Anal. Calcd for C_26_H_17_F_3_N_4_O: C, 68.12; H, 3.74; N, 12.22; Found: C, 67.94; H, 3.97; N, 12.08.

N'*-Benzylidene-1-(4-chlorophenyl)-β-carboline-3-carbohydrazide* (C_25_H_17_ClN_4_O, m.w. 424.9) (**c4**). Yield: 86%; m.p.: 220–221 °C; FAB-MS *m/z* (M+1) 425; IR (cm^−1^): 3419, 1683, 1624, 1511, 1351; ^1^H-NMR: 7.34 (1H, q, *J* = 15.6 Hz, C(6)H, c), 7.46 (3H, m, *J* = 30.6 Hz, C(3,4,5)H, Ph2), 7.62 (1H, m, *J* = 16.2 Hz, C(7)H, c), 7.70 (1H, d, *J* = 8.4 Hz, C(8)H, c), 7.73 (2H, d, *J* = 15.6 Hz, C(3,5)H, Ph1), 7.77 (2H, m, *J* = 8.4 Hz, C(2,6)H，Ph2), 8.26 (2H, d, *J* = 9 Hz, C(2,6)H, Ph1), 8.48 (1H, d, *J* = 8.4 Hz, C(5)H, c), 8.68 (1H, s, C(4)H, c), 8.98 (1H, s, –N=CH), 11.83 (1H, s, –NH, c), 11.97 (1H, s, –NH–N); ^13^C-NMR: 112.7, 114.9, 120.3, 121.6, 122.9, 124.3, 128.1, 129.4, 130.5, 131.7, 132.8, 134.2, 135.6, 138.5, 139.6, 141.6, 144.8, 148.1, 148.5, 160.3; Anal. Calcd for C_25_H_17_ClN_4_O: C, 70.67; H, 4.03; N, 13.19; Found: C, 70.42; H, 4.29; N, 13.05. 

N'*-(4-Trifluoromethylbenzylidene)-1-(4-trifluoromethyl-phenyl)-β-carboline-3-carbohydrazide)* (C_27_H_16_F_6_N_4_O, m.w. 526.4) (**d1**). Yield: 83.9%, m.p.: 289–290 °C; FAB-MS *m/z* (M+1) 527; IR (cm^−1^): 3438, 1735, 1658, 1381; ^1^H-NMR: 7.34–8.48 (12H, Ph_1_, Ph_2_, c), 8.75 (1H, s, C(4)H, c), 8.99 (1H, s, –N=CH), 12.02 (1H, s, –NH, c), 12.09 (1H, s, –NH–N); ^13^C-NMR: 112.7, 114.7, 120.3, 121.5, 122.9, 124.3, 127.3, 128.8, 129.1, 131.2, 132.7, 138.5, 144.6, 147.3, 148.5, 149.2, 150.9, 161.8; Anal. Calcd for C_27_H_16_F_6_N_4_O: C, 61.60; H, 3.06; N, 10.64; Found: C, 61.38; H, 3.24; N, 10.47. 

N'*-(4-Trifluoromethylbenzylidene)-1-phenyl-β-carboline-3-carbohydrazide* (C_26_H_17_N_4_OF_3_, m.w. 458.4) (**d2**). Yield: 86.6%, m.p.: 235–237 °C; FAB-MS *m/z* (M+1) 459; IR (cm^−1^): 3379, 1723, 1594, 1487, 1364; ^1^H-NMR: 7.33–8.76 (13H, Ph_1_, Ph_2_, c), 8.98 (1H, s, C(4)H, c), 9.00 (1H, s, –N=CH), 11.98 (1H, s, –NH, c), 11.99 (1H, s, –NH–N); ^13^C-NMR: 112.5, 114.9, 120.4, 121.8, 122.5, 125.2, 127.3, 128.1, 129.3, 130.5, 131.2, 131.8, 134.7, 135.6, 138.5, 139.6, 142.6, 145.2, 148.3, 148.5, 160.2; Anal. Calcd for C_26_H_17_F_3_N_4_O: C, 68.12; H, 3.74; N, 12.22; Found: C, 67.96; H, 3.97; N, 12.11.

N'*-(4-Trifluoromethylbenzylidene)-1-(4-chlorophenyl)-β-carboline-3-carbohydrazide* (C_26_H_16_ClN_4_OF_3_, m.w. 492.9) (**d3**). Yield: 88.8%, m.p.: 140–142 °C; FAB-MS *m/z* (M+1) 493; IR (cm^−1^): 3427, 1692, 1583, 1491; ^1^H-NMR: 7.36–8.76 (12H, Ph_1_, Ph_2_, c), 8.76 (1H, s, C(4)H, c), 8.99 (1H, s, –N=CH), 12.02 (1H, s, –NH, c), 12.03 (1H, s, –NH–N); ^13^C-NMR: 112.7, 115.1, 120.6, 121.5, 121.9, 124.7, 127.2, 128.4, 129.5, 132.2, 132.7, 138.5, 144.6, 147.3, 148.2, 148.9, 150.4, 160.6; Anal. Calcd for C_26_H_16_ClF_3_N_4_O: C, 63.36; H, 3.27; N, 11.37; Found: C, 63.19; H, 3.45; N, 11.24.

### 3.2. Biological Assays

All compounds were formulated as 10 g/L emulsified concentrates using DMSO as solvent and TW-80 as emulsification reagent. The stock solutions were diluted with water to the required concentration and applied to pot-grown plants in a greenhouse at 23 ± 1 °C, 60 ± 5% relative humidity (RH), 10 flux light intensity and 8 h/day photoperiod. Twenty seeds of each weed species including Chinese cabbage (rape, *Brassica campestris *L. ssp*.*) and barnyard grass (*Echinochloa crusgalli*) were chosen for testing. Seedlings were grown in the test plate of 9-cm diameter containing two pieces of filter paper and 9 mL solution of the tested compound (100 mg/L and 10 mg/L, respectively). Distilled water and 2,4-D were used as the comparison compounds. The herbicidal activity was assessed by the inhibitory rate in comparison with the distilled water. The heights of the above-ground parts of the seedlings, their root lengths and fresh weights in each cup were measured, and the means were calculated. The percentage inhibition was used to describe the control efficiency of the compounds. Range from 0 to 100%, 0% means no effect and 100% means inhibition. The test was repeated three times; means were calculated and shown as activity in Table 1. The compounds with higher activity were selected for further investigations of EC50 and probit (mortality%)-log (concentration) lines, which are shown in Table 2.

## 4. Conclusions

New 1,2,3,4-tetrahydro-β-carboline, β-carboline and 1-substituted-β-carboline derivatives bearing a substituted carbohydrazide group at C-3 were designed and synthesized to study the structure-activity relationships of their analogues. The results showed that some of the newly synthesized carbohydrazide derivatives carrying phenyl groups substituted with an electron-withdrawing substituent could be used for the further development of novel herbicides. 
